# Determinants of Developing Stroke Among Low-Income, Rural Residents: A 27-Year Population-Based, Prospective Cohort Study in Northern China

**DOI:** 10.3389/fneur.2019.00057

**Published:** 2019-02-05

**Authors:** Yanan Wu, Zhenqian Fan, Yu Chen, Jingxian Ni, Jie Liu, Jing Han, Li Ren, Jun Tu, Xianjia Ning, Jinghua Wang

**Affiliations:** ^1^Department of Neurology, Tianjin Medical University General Hospital, Tianjin, China; ^2^Laboratory of Epidemiology, Tianjin Neurological Institute, Tianjin, China; ^3^Key Laboratory of Post-Neuroinjury Neuro-Repair and Regeneration in Central Nervous System, Tianjin Neurological Institute, Ministry of Education and Tianjin City, Tianjin, China; ^4^Department of Endocrinology and Metabolism, The Second Hospital of Tianjin Medical University, Tianjin, China

**Keywords:** stroke, risk factors, epidemiology, population-based study, cohort study

## Abstract

Although strokes are the leading cause of death and disability in many countries, China still lacks long-term monitoring data on stroke incidence and risk factors. This study explored stroke risk factors in a low-income, rural population in China. The study population was derived from the Tianjin Brain Study, a population-based stroke monitoring study that began in 1985. This study documented the demographic characteristics, past medical histories, and personal lifestyles of the study participants. In addition, physical examinations, including measurements of blood pressure (BP), height, and weight, were performed. Hazard ratios (HRs) were estimated for the risk factors for all subtypes of stroke using multivariate Cox regression analyses. During the study with mean following-up time of 23.16 years, 3906 individuals were recruited at baseline, and during 27 years of follow-up, 638 strokes were documented. The multivariate Cox regression analyses revealed a positive correlation between age and stroke incidence. Limited education was associated with a 1.9-fold increase in stroke risk (lowest vs. highest education level). Stroke risk was higher among former smokers than among current smokers (HR, 1.8 vs. 1.6; both, *P* < 0.05). Moreover, stroke risk was significantly associated with sex (HR, 1.8), former alcohol drinking (HR, 2.7), baseline hypertension (HR, 3.1), and overweight (HR, 1.3). In conclusion, this study identified uncontrollable (sex and age) and controllable (education, smoking, alcohol drinking, hypertension, and overweight) risk factors for stroke in a low-income, rural population in China. Therefore, it is critical to control BP and weight effectively, advocate cessation of smoking/alcohol drinking, and enhance the education level in this population to prevent increase in the burden of stroke in China.

## Introduction

Cerebrovascular disease is one of the leading causes of death and disability worldwide (1, 2). Stroke, a type of cerebrovascular disease, has been reported by the World Health Organization as the leading cause of disability among adults and the second leading cause of death worldwide (3). Overall, 71% of stroke-related deaths and 78% of disability-adjusted life loss occurred in low- and middle-income countries (4). In China, strokes account for 21.6% of total male mortality and 20.8% of total female mortality. Thus, strokes remain a major health problem in China (5).

Over the past two decades, China has experienced rapid health and sociodemographic changes that have affected the prevalence of common risk factors for stroke (6, 7). Although developed countries have shown a significant downward trend in stroke incidence through effective control of risk factors (8–10), China still lacks long-term monitoring data on stroke incidence and risk factors, especially among rural residents with low income and poor access to education. Therefore, this study aimed to explore the risk factors for developing stroke among the low-income, rural population in northern China.

## Materials and Methods

### Study Population and Sample Process

This is a population-based cohort study, which has conducted since 1991 in rural Tianjin, China. The study population has been described previously (11–14). Briefly, it included 14,936 people in 1991, 95% of whom were low-income farmers living in 18 administrative villages. The main source of income was cereal crop production, and the per capita incomes were <100 USD in 1990 and <1000 USD in 2010 (15).

The sampling method used in this cohort study has also been reported previously (16). In short, all residents living in the 18 administrative villages were recruited into the study. The villages were divided into three geographic regions (east, south, and north), and two villages were randomly selected from each region. Using a stratified cluster sampling approach, we selected all residents (≥15 years old) from the six villages who had no histories of ischemic heart disease (such as coronary heart disease or myocardial infarction) or stroke as participants of the survey. For this analysis, only participants aged ≥18 years were included.

This study was carried out in accordance with the recommendations of the Study involving human subjects, committee of Tianjin Medical University General Hospital. All subjects gave written informed consent in accordance with the Declaration of Helsinki. The protocol was approved by the ethics committee of Tianjin Medical University General Hospital.

### Baseline Information

All information regarding demographic characteristics (including sex, age, and education level), past medical histories (including hypertension, diabetes mellitus [DM], stroke, and cardiovascular disease), and personal lifestyles (including smoking and alcohol drinking) was collected by trained local researchers through face-to-face interviews. Physical examinations, which included measurements of blood pressure (BP), height, and weight, were conducted during the interview.

### Measurement Methods

The BP measurement methods are described previously (16). Briefly, standardized BP measurements were performed using a mercury sphygmomanometer, with the cuff adjusted to each patient's arm circumference. BP was measured by placing the cuff on the arm, at the level of the heart. If the difference between two systolic BP (SBP) readings was <10 mmHg and/or the difference between two diastolic BP (DBP) readings was <5 mmHg, the average of the two measurements was recorded, with the latter measurement taken after the patient had rested for 5 min in the supine position. If the differences between the two readings were outside of the indicated ranges, or the BP reached the criterion for hypertension, another two readings were obtained after an additional 20 min of resting. Hypertension was categorized into four types: normal BP, isolated systolic hypertension (ISH), isolated diastolic hypertension (IDH), and systolic-diastolic hypertension (SDH).

Body mass index (BMI) was calculated as weight (in kilograms) divided by the square of height (in meters), and categorized as follows: normal (<24 kg/m^2^), overweight (24–27.9 kg/m^2^), and obese (≥28 kg/m^2^) (17).

### Case Definition

The stroke monitoring details and protocol used to diagnose the type of stroke are described in our previous reports (11–14). Stroke was defined as an acute, focal, neurological deficit with vascular etiology that lasts for >24 h. Stroke events included the ischemic stroke (IS) and hemorrhagic stroke subtypes. Hemorrhagic stroke was defined as an intracerebral hemorrhage (ICH) or subarachnoid hemorrhage; IS was defined as a thrombotic brain infarction, cardioembolic stroke, or lacunar infarct. Undefined strokes were those that could not be classified as either of the subtypes (18). The stroke subtypes were identified using neuroimaging examination (computed tomography or magnetic resonance imaging). All included stroke events were those diagnosed as full clinical strokes, demonstrating obvious clinical signs and symptoms. Transient ischemic attacks (TIAs) and silent strokes (diagnosed using imaging only) were excluded, and strokes occurring in individuals with histories of TIAs were considered as incident events. Patients with transient symptoms but with neuroimaging evidence of cerebral infarctions were considered as stroke cases (19). During the early phase of this study (1992–1998), strokes were confirmed mainly based on clinical examinations by senior neurologists for nonhospitalized patients and on medical records for hospitalized patients.

### Statistical Analysis

Continuous variables (age, BP, and BMI) were expressed as means (standard deviation), and categorical variables were expressed as 95% confidence intervals (CIs). Age-standardized incidences were calculated using the direct method and standard population age groups: < 35, 35–39, 40–44, 45–49, 50–54, 55–59, 60–64, 65–69, 70–74, and ≥75 years (20). Subgroup analyses were conducted to evaluate the risk of first-ever stroke by age (<35, 35–44, 45–54, 55–64, 65–74, or ≥75 years), education level (illiterate, 1–6 years of schooling, 7–9 years, or >9 years), SBP (<130, 130–139, 140–159, 160–179, or ≥180 mmHg), DBP (<80, 80–89, 90–99, 100–109, or ≥110 mmHg), hypertension type (normal BP, ISH, IDH, or SDH), BMI (normal, overweight, or obese), smoking status (never smoked, former smoked, or currently smoked), and alcohol drinking status (never drank, former drank, or currently drinks). The determinants of all stroke subtypes (“total stroke” in succeeding instances) were estimated using Cox regression analysis; the results were presented as adjusted HR and 95% CIs after adjustment for covariates (age, sex, hypertension, diabetes, body mass index [BMI], hypertension types, current smoking and alcohol consumption status). In this model, there were two outcome variables (status and survival time), they were the dependent variables. Status was a dichotomous variable which was categorized stroke group and non-stroke group. Survival time, which was a continuous variable, was defined as the interval time from baseline to onset time for those participants with first-ever stroke, from baseline to June 2018 for those survivals without developing stroke (censored data) and from baseline to death date for those dead participants (censored data). All associated covariates assessed using Kaplan-Meier test were performed the Cox regression analysis. The assumption of model was that the impact of risk factors on occurring stroke events did not vary with survival time. All statistical analyses were performed using SPSS for Windows (version 19.0; SPSS, Chicago, IL, United States). A *P* < 0.05 was considered statistically significant.

## Results

### Demographic Characteristics of Participants

This study recruited 3,906 individuals (men, 1,834 [47.0%]; women, 2,072 [53.0%]; mean age, 41.74 ± 16.58 years) at baseline, with mean following-up time of 23.16 years. During 27 years of follow-up (total, 85,346.5 person-years), a total of 638 strokes (379 [59.4%] in men and 259 [40.6%] in women) were documented, including 404 ISs (63.3%), 121 hemorrhagic strokes (19.0%), and 113 undefined strokes (17.7%). The stroke incidence per 100,000 person-years in this population was as high as 748 (473 for IS; 142 for ICH). The incidence of total stroke was significantly higher among men than among women (978 vs. 556 for total stroke; 596 vs. 371 for IS; 206 vs. 88 for ICH).

The education level of the participants was very low, with 40.7% of them (men, 42.5%; women, 57.5%) having never received any formal education. The baseline prevalence was 31.0% for hypertension, 0.1% for DM, 25.7% for current smoking, and 15.4% for current alcohol drinking. The average SBP, DBP, and BMI values were 127.39 mmHg, 79.95 mmHg, and 22.58 kg/m^2^), respectively (Table 1).

**Table 1:** Demographical characteristics of all participants in this study.

**Risk factors**	**Total**	**Men**	**Women**
Total:	3,906	1,834 (47.0)	2,072 (53.0)
Age, means (SD), years	41.74 (16.58)	42.51(16.89)	41.07 (16.28)
Age group, n (%)			
< 35 years	1,582	720 (45.5)	862 (54.5)
35 44 years	906	401 (44.3)	505 (55.7)
45 54 years	466	238 (51.1)	228 (48.9)
55 64 years	470	222 (47.2)	248 (52.8)
65 74 years	320	168 (52.5)	152 (47.5)
≥75 years	162	85 (52.5)	77 (47.5)
Education, n (%)			
0 years	1,588	675 (42.5)	913 (57.5)
1 6 years	978	508 (51.9)	470 (48.1)
7 9 years	1,203	584 (48.5)	619 (51.5)
> 9years	137	67 (48.9)	70 (51.5)
Smoking status, n (%)			
Current smoking	1,002	921 (91.9)	81 (8.1)
Former smoking	112	101 (90.2)	11 (9.8)
Never smoking	2,792	812 (29.1)	1,980 (70.9)
Alcohol consumption, n (%)			
Current drinking	602	577 (95.8)	25(4.2)
Former drinking	16	16 (100)	0
Never drinking	3,288	1,241 (37.7)	2,047(62.3)
SBP, means (SD), mmHg	127.39 (20.27)	127.92 (17.57)	126.92 (22.39)
DBP, means (SD), mmHg	79.95 (11.41)	80.60 (10.59)	79.37 (12.06)
Baseline Hypertension, n (%)			
No	2,695	1,250 (46.4)	1,445 (53.6)
Yes	1211	584 (48.2)	627 (51.8)
BP types, n (%)			
Normal BP	2,708	1,257 (46.4)	1,451 (53.6)
ISH	177	85 (48.0)	92 (52.0)
IDH	328	182 (55.5)	146 (44.5)
SDH	693	310 (44.7)	383 (55.3)
Baseline Diabetes, n (%)			
No	3,902	1,834 (47.0)	2,068 (53.0)
Yes	4	0	4(100)
BMI, means (SD), Kg/m^2^	22.58 (2.80)	22.32 (2.37)	22.83 (3.11)
BMI groups, n (%)			
Normal weight	2,897	1,466 (50.6)	1,431 (49.4)
Overweight	847	332 (39.2)	515 (60.8)
Obesity	162	36 (22.2)	126 (77.8)

*SD, standard deviation; BMI, body mass index; SBP, systolic blood pressure; DBP, diastolic blood pressure*.

### Associated Factors for Developing Stroke in the Univariate Analysis

During the follow-up period, the average age at baseline was higher among individuals with stroke (52.4 ± 14.7 years for total stroke; 49.5 ± 13.5 years for IS; 49.6 ± 15.1 years for ICH) than among those without stroke (all *P* < 0.05). Simultaneously, the incidences of total stroke and ICH increased with increasing age at baseline among individuals aged <75 years, and the incidences of ICH decreased with increasing education level (*P* < 0.05). The incidences were higher in ever-smokers (32.1% for total stroke; 25.9% for IS; 4.5% for ICH) and those with hypertension (27.0% for total stroke; 15.9% for IS; 4.7% for ICH; *P* < 0.05) than in those who never smoked or had no hypertension. However, stroke incidence was highest among former alcohol drinkers, except for ICH. Additionally, the incidence of total stroke in patients with DM (50.0%) at baseline was significantly higher than that in patients without DM (16.3%, *P* < 0.05). In terms of hypertension type, the incidence of total stroke was highest in patients with SDH, followed by those with ISH and IDH. Stroke incidence was also greater among overweight or obese participants than among normal-weight participants (Table 2).

**Table 2:** Associated factors of developing stroke in the univariate analysis.

**Risk factors**	**Without stroke (*n* = 3,268)**	**Stroke (*n* = 638)**	**IS (*n* = 404)**	**ICH (*n* = 121)**
Age, means (SD), years	39.7(16.1)	52.4 (14.7)	49.5 (13.5)^†^	49.6 (15.1)^†^
Age group, n (%)		^*^	^*^	^*^
< 35 years	1,495 (94.5)	87 (5.5)	63 (4.0)	24 (1.5)
35 44 years	785 (86.6)	121 (13.4)	95 (10.5)	22 (2.4)
45 54 years	353 (75.8)	113 (24.2)	80 (17.2)	23 (4.9)
55 64 years	308 (65.5)	162 (34.5)	108 (23.0)	28 (6.0)
65 74 years	198 (61.9)	122 (38.1)	49 (15.3)	21 (6.6)
≥75 years	129 (79.6)	33 (20.4)	9 (5.6)	3 (1.9)
Sex, (%)		^*^	^*^	^*^
Male	1,455 (79.3)	379 (20.7)	231 (12.6)	80 (4.4)
Female	1,813 (87.5)	259 (12.5)	173 (8.3)	41 (2.0)
Education, n (%)		^*^	^*^	^*^
0 years	1,246 (78.5)	342 (21.5)	191 (12.0)	68 (4.3)
1 6 years	786 (80.4)	192 (19.6)	128 (13.1)	39 (4.0)
7 9 years	1,105 (91.9)	98 (8.1)	80 (6.7)	13 (1.1)
> 9years	131 (95.6)	6 (4.4)	5 (3.6)	1 (0.7)
Smoking status, n (%)		^*^	^*^	^*^
Current smoking	802 (80.0)	200 (20.0)	124 (12.4)	42 (4.2)
Former smoking	76 (67.9)	36 (32.1)	29 (25.9)	5 (4.5)
Never smoking	2,390 (85.6)	402 (14.4)	251 (9.0)	74 (2.7)
Alcohol consumption, n (%)		^*^	^*^	
Current drinking	482 (80.1)	120 (19.9)	83 (13.8)	19 (3.2)
Former drinking	7 (43.8)	9 (56.3)	7 (43.8)	2 (12.5)
Never drinking	2,779 (84.5)	509 (15.5)	314 (9.5)	100 (3.0)
Baseline hypertension, n (%)		^*^	^*^	^*^
No	2384 (88.5)	311 (11.5)	212 (7.9)	64 (2.4)
Yes	884 (73.0)	327 (27.0)	192 (15.9)	57 (4.7)
SBP, means (SD), mmHg				
	125.5 (19.0)	137(23.7)^†^	134.0 (22.0)^†^	137.1 (24.4)^†^
DBP, means (SD), mmHg				
	78.9 (10.8)	85.2(13.0)^†^	84.0 (12.2)^†^	85.6 (14.1)^†^
BP type, n (%)		^*^	^*^	^*^
Normal	2,393 (88.4)	315 (11.6)	215 (7.9)	64 (2.4)
ISH	138 (78.0)	39 (22.0)	21 (11.9)	6 (3.4)
IDH	270 (82.3)	58 (17.7)	43 (13.1)	9 (2.7)
SDH	467 (67.4)	226 (32.6)	125 (18.0)	42 (6.1)
Baseline diabetes, n (%)		^*^		
No	3,266 (83.7)	636 (16.3)	403 (10.3)	121 (3.1)
Yes	2 (50.0)	2 (50.0)	1 (25.0)	0
BMI, means(SD), Kg/m^2^				
	22.5 (2.8)	23.2 (3.0)^†^	23.3 (2.8)^†^	23.3 (2.8)^†^
BMI groups, n (%)		^*^	^*^	^*^
Normal weight	2,483 (85.7)	414 (14.3)	261 (9.0)	71 (2.5)
Overweight	664 (78.4)	183 (21.6)	113 (13.3)	45 (5.3)
Obesity	121 (74.7)	41 (25.3)	30 (18.5)	5 (3.1)

*SD, standard deviation; SBP, systolic blood pressure; DBP, diastolic blood pressure; ISH, isolated systolic hypertension; IDH, isolated diastolic hypertension; SDH, systolic-diastolic hypertension; BMI, body mass index*.

† *Presented P < 0.05 comparing to individuals without stroke*.

* *Presented P < 0.05 in the different groups*.

### Determinants of Developing Stroke in the Multivariate Analysis

In the Cox proportional hazards model, a positive correlation was noted between age and stroke incidence. Compared with the <35-year-old group, participants aged ≥75 years had the highest risk of onset (HR, 23.9; 95% CI, 15.5–36.8; *P* < 0.001). Education level was a protective factor for stroke onset, but the stroke risk for those with 7–9 years of formal education was not any higher than the stroke risk for those with >9 years of education (P>0.05). Although smoking was also a risk factor for stroke, the stroke risk among ever-smokers was higher than that among current smokers (HR, 1.8 vs. 1.6; all *P* < 0.05). Stroke risk was also significantly associated with sex (HR, 1.8), previous alcohol drinking (HR, 2.7), baseline hypertension (HR, 3.1), and overweight (HR, 1.3; Table 3).

**Table 3:** Determinants of developing stroke in the multivariate analysis.

**Effected factors**	**Reference**	**HR (95%CI)**	***P***
Men	Women	1.8 (1.5, 2.1)	< 0.001
Age	< 35 years		
35 44 years		2.4 (1.8, 3.2)	< 0.001
45 54 years		4.7 (3.5, 6.3)	< 0.001
55 64 years		9.7 (7.2, 12.9)	< 0.001
65 74 years		18.4 (13.5, 25.1)	< 0.001
≥75 years		23.9 (15.5, 36.8)	< 0.001
Education	>9 years		
0 years		2.9 (1.3, 6.5)	0.011
1~6 years		3.1 (1.4, 7.0)	0.007
7~9 years		2.2 (1.0, 5.1)	0.059
Smoking	Never smoking		
Current smoking		1.6 (1.3, 1.9)	< 0.001
Former smoking		1.8 (1.2, 2.7)	0.002
Drinking	Never drinking		
Current drinking		0.9 (0.7, 1.1)	0.335
Former drinking		2.7 (1.3, 5.4)	0.007
Baseline hypertension	No		
Yes		3.1 (1.1, 8.3)	0.026
Baseline diabetes	No		
Yes		3.7 (0.9, 15.0)	0.066
BP type	Normal		
ISH		1.0 (0.4, 2.8)	0.996
IDH		0.2 (0.2, 1.4)	0.198
SDH		0.4 (1.5, 0.6)	0.434
BMI groups	Normal weight		
Overweight		1.3 (1.1, 1.5)	0.006
Obesity		1.3 (0.9, 1.8)	0.105

*ISH, isolated systolic hypertension; IDH, isolated diastolic hypertension; SDH, systolic-diastolic hypertension*.

### Determinants of Stroke by Stroke Subtype

The risk of IS was higher among men than among women (HR, 1.90; 95% CI, 1.48–2.43; *P* < 0.001). Compared with the <35-year-old group, participants aged 65–74 years had the highest risk of stroke (HR, 9.60; 95% CI, 6.24–14.78; *P* < 0.001). The risk of IS was also significantly associated with current alcohol drinking (HR, 0.69; 95% CI, 0.52–0.93), former alcohol drinking (HR, 2.51; 95% CI, 1.13–5.62), overweight (HR, 1.33; 95% CI, 1.06–1.67), and obesity (HR, 1.91; 95% CI, 1.29–2.82; Table 4).

**Table 4:** Determinants of developing ischemic stroke in the multivariate analysis.

**Effected factors**	**Reference**	**HR (95%CI)**	***P***
Men	Women	1.90 (1.48,2.43)	< 0.001
Age	< 35 years		
35 44 years		2.57 (1.85,3.58)	< 0.001
45 54 years		4.40 (3.07,6.30)	< 0.001
55 64 years		8.38 (5.81,12.07)	< 0.001
65 74 years		9.60 (6.24,14.78)	< 0.001
≥75 years		8.46 (3.97,18.03)	< 0.001
Education	>9 years		
0 years		2.2 (0.9, 5.4)	0.082
1~6 years		2.6 (1.0, 6.3)	0.052
7~9 years		2.1 (0.9, 5.2)	0.105
Smoking	Never smoking		
Current smoking		1.07 (0.82,1.39)	0.615
Former smoking		1.17 (0.76,1.81)	0.476
Drinking	Never drinking		
Current drinking		0.69 (0.52,0.93)	0.013
Former drinking		2.51 (1.13,5.62)	0.025
Baseline hypertension	No		
Yes		2.40 (0.74,7.81)	0.146
BP type	Normal		
ISH		0.52 (0.15,1.81)	0.303
IDH		0.57 (0.17,1.92)	0.366
SDH		0.85 (0.26,2.78)	0.788
BMI groups	Normal weight		
Overweight		1.33 (1.06,1.67)	0.013
Obesity		1.91 (1.29,2.82)	0.001

*ISH, isolated systolic hypertension; IDH, isolated diastolic hypertension; SDH, systolic-diastolic hypertension*.

Meanwhile, the risk of ICH was higher in men than in women (HR, 2.88; 95% CI, 1.86–4.47; *P* < 0.001). Compared with the <35-year-old group, participants aged 65–74 years had the highest risk of onset (HR, 5.12; 95% CI, 2.60–10.09; *P* < 0.001). Education level was a protective factor for ICH onset, but the risk of ICH in those with 7–9 years of formal education was statistically significant (*P* < 0.001). The risk of ICH was also significantly associated with overweight (HR, 2.18; 95% CI, 1.48–3.20; Table 5).

**Table 5:** Determinants of developing hemorrhagic stroke in the multivariate analysis.

**Effected factors**	**Reference**	**HR(95%CI)**	***P***
Men	Women	2.88 (1.86,4.47)	< 0.001
Age	< 35 years		
35 44 years		1.20 (0.66,2.18)	0.551
45 54 years		2.36 (1.28,4.33)	0.006
55 64 years		3.11 (1.68,5.77)	< 0.001
65 74 years		5.12 (2.60,10.09)	< 0.001
≥75 years		3.87 (1.08,13.92)	0.038
Education	>9 years		
0 years		5.0 (0.7, 36.7)	0.111
1~6 years		4.6 (0.6, 33.8)	0.333
7~9 years		1.6 (0.2, 12.7)	0.629
Smoking	Never smoking		
Current smoking		0.97 (0.63,1.50)	0.898
Former smoking		0.55 (0.21,1.40)	0.207
Baseline hypertension	No		
Yes		969.49 (0.00,6.85)	0.886
BP type	Normal		
ISH		945.73 (0.00,6.70)	0.886
IDH		931.11 (0.00,6.59)	0.887
SDH		2034.18 (0.00,1.44)	0.874
BMI groups	Normal weight		
Overweight		2.18 (1.48,3.20)	< 0.001
Obesity		1.38 (0.55,3.48)	0.499

*ISH, isolated systolic hypertension; IDH, isolated diastolic hypertension; SDH, systolic-diastolic hypertension*.

## Discussion

To our knowledge, this is the first prospective study to explore the comprehensive risk factors for stroke among low-income and poorly educated individuals in China. In this study, the overall stroke incidence per 100,000 person-years in this population was as high as 748 (473 for IS; 142 for ICH). For total stroke, sex, age, smoking status, alcohol drinking status, baseline hypertension, and overweight were risk factors, while education level was a protective factor. For IS, sex, age, former alcohol drinking, overweight, and obesity were risk factors, whereas current alcohol drinking was a protective factor. For ICH, sex, age, and overweight were risk factors, while an education level of >9 years was a protective factor.

Previous studies have reported a dramatic increase in the incidence of first-ever stroke. The age-standardized incidence of first-ever stroke per 100,000 person-years increased rapidly from 124.5 in 1992–1998 to 190.0 in 1999–2005 and to 318.2 in 2006–2012; overall, the incidence increased by 6.5% annually for this population (11). The incidences of total stroke were 586.8 per 100,000 person-years in northeastern Greece, 76.5 per 100,000 person-years in Argentina, and 345.1 per 100,000 person-years in China (21–23). However, in our study, the stroke incidence was as high as 748 per 100,000 person-years. The higher prevalence of risk factors for stroke, including hypertension, obesity, smoking, and alcohol drinking, in this population may have contributed to the elevated incidence of stroke (11).

The influence of sex and age on stroke incidence is being increasingly recognized and evaluated. With advancing age, the stroke incidence for both men and women increases exponentially (24), with approximately 75–89% of strokes occurring in individuals aged >65 years (25), consistent with our findings. Compared with individuals aged <35 years, stroke risk was 23.9 times higher among individuals aged ≥75 years, and participants aged 65–74 years had the highest risk of onset of IS and ICH. A study from Sweden also indicated that stroke risk was significantly associated with age (risk ratio [RR] per 1 year of age, 1.12) in individuals with normal BP (26).

Our results showed that stroke risk in men was 1.8 times higher than that in women. Similarly, a Swiss study showed a higher age-specific stroke incidence in men than in women (27). Overall, the global age-adjusted incidence ratio for strokes in men vs. women is 1.33, with the highest ratio occurring among individuals aged of 35–44 years and decreasing after the age of 75 years (28). The excess rate of stroke in women of advanced age may arise from their longer life expectancy than men, which results in increasing age being associated with higher stroke risk (24, 29). However, in our study population, further study is required to investigate the sex-specific differences in age-related stroke incidence. This may be partially explained by studies that have reported that estrogens beneficially affect the vasculature by improving endothelial function, enhancing vasodilation, and increasing blood flow after vascular occlusion; estrogens also have antioxidative and anti-inflammatory effects (30) and inhibit platelet aggregation (31).

Besides sex and age, smoking and alcohol drinking are independent risk factors for stroke (32–36). Although a previous studies failed to show a significant increase in total stroke risk among ever-smokers compared with never-smokers (37–39), a study from Japan found an 18% decrease in total stroke risk within the first 2 years after smoking cessation, with the maximum effect (38% risk reduction) occurring 2–4 years after smoking cessation (32). Our results also showed that smoking was a risk factor for stroke, and stroke risk was higher among former smokers than among current smokers (HR, 1.8 vs. 1.6). The reason may be because this low-education population only quit smoking after they developed a serious disease.

Previous meta-analyses (40, 41) also suggested that heavy alcohol drinking is associated with an increased risk of stroke, but that low-to-moderate intake may be protective against total stroke and IS. In this study, there was a significant increase in the risk of total stroke and IS among those who were former alcohol drinkers, but there was a significant decrease in risk among those who currently drink compared with those who never drank. This may be because low-dose alcohol drinking among current drinkers may have protective effects against stroke. Therefore, further study is needed to quantify the amount of alcohol consumed by this study population.

In our study population, ever-smokers and ever-drinkers often experienced diseases such as hypertension, and we did not have access to statistics regarding the durations of smoking and drinking cessation. Hypertension was more prevalent among ever-smokers than among never-smokers or current smokers (32). Previous studies have shown that alcohol drinking is also associated with elevated BP (42). Hypertension is often reported to be the most common and strongest risk factor associated with strokes. In a Chinese study (43) of five stroke-associated risk factors (hypertension, dyslipidemia, obesity, diabetes, and smoking), hypertension was most strongly associated with stroke risk. The results of the present study also indicated that baseline hypertension was a strong risk factor for stroke (HR, 3.1).

Diabetes is another classic risk factor for stroke. In our analysis, the prevalence of diabetes was significantly higher among stroke patients, but the relationship became nonsignificant in the multivariate analysis. This may have been due to the very low number of patients (*n* = 4) who exhibited known diabetes at baseline. The awareness, treatment, and control rates for diabetes in this population were 42.9, 11.1, and 11.1% in 1992, respectively. The low level of knowledge about diabetes due to the low income and low education status in this population may be the main cause of the lower prevalence of diabetes in this study.

Comparing the lowest and highest education levels, a study has demonstrated that low education level was associated with a 2.5-fold increase in stroke risk (44). Similarly, a Swedish study involving middle-aged women reported an elevated stroke risk among those with less education. In that study, the associated excess risk was largely attributed to lifestyle and biological risk factors for stroke, particularly smoking and alcohol drinking (45). Consistent with these earlier observations, the findings of the present study showed that education was a protective factor for total stroke and ICH.

A previous meta-analysis reported an RR of 1.22 (95% CI, 1.05–1.41) for IS in overweight patients and an RR of 1.64 (95% CI, 1.36–1.99) for IS in obese patients (46). Additionally, multivariate analyses of data from a study involving 76,227 Chinese adults showed that a 2 kg/m^2^ increase in baseline BMI increased the total stroke RR by 6.1% (47). Similarly, in the present study, overweight and obesity increased the risk of developing IS. Moreover, overweight was associated with a high risk of stroke and ICH.

This study has some limitations. The study population was selected from a township in northern China that was not representative of the country's overall population. Although this study was a prospective cohort study involving a rural, low-income population, a larger population and a longer study period may have reduced the impact of the limited representativeness of the sample on the study results. Furthermore, we did not collect information regarding all the medicines used by the participants. However, because of their low socioeconomic status, the frequency of medicine use in this population was low (16) and may not significantly affect the validity of the results.

## Conclusions

This study identified uncontrollable (sex and age) and controllable (education, smoking, alcohol drinking, hypertension, and overweight) risk factors for stroke in a low-income, rural population in China. Therefore, it is critical for clinical doctors to control BP and weight effectively, advocate cessation of smoking/alcohol drinking, and enhance the education level in this population to prevent increase in the burden of stroke.

## Author Contributions

JW and XN: contributed to the conception and design of the work; YW, ZF, YC, JN, JL, JH, LR, and JT: contributed the data acquisition; JW and XN: contributed the analysis and interpretation of data for the work; YW, ZF, and YC: contributed drafting the work; JW and XN: contributed revising the work for important intellectual content. All authors approved of the final version to be published, and agree to be accountable for all aspects of the work in ensuring that questions related to the accuracy or integrity of any part of the work are appropriately investigated and resolved.

### Conflict of Interest Statement

The authors declare that the research was conducted in the absence of any commercial or financial relationships that could be construed as a potential conflict of interest.
